# Examining dyslipidaemia, metabolic syndrome and liver enzyme levels in patients with prediabetes and type 2 diabetes in population from Hoveyzeh cohort study: A case–control study in Iran

**DOI:** 10.1002/edm2.401

**Published:** 2023-01-03

**Authors:** Negar Dinarvand, Bahman Cheraghian, Zahra Rahimi, Samaneh Salehipour Bavarsad, Amirhooshang Bavarsad, Narges Mohammadtaghvaei

**Affiliations:** ^1^ Hyperlipidemia Research Center Ahvaz Jundishapur University of Medical Sciences Ahvaz Iran; ^2^ Alimentary Tract Research Center, Department of Biostatistics & Epidemiology School of Public Health Ahvaz Jundishapur University of Medical Sciences Ahvaz Iran; ^3^ Department of Biostatistics and Epidemiology School of Public Health Ahvaz Jundishapur University of Medical Sciences Ahvaz Iran; ^4^ Alimentary Tract Research Center, Department of Internal Medicine, School of Medicine Ahvaz Jundishapur University of Medical Sciences Ahvaz Iran; ^5^ Department of Laboratory Sciences, Faculty of Paramedicine Ahvaz Jundishapur University of Medical Sciences Ahvaz Iran

**Keywords:** Hoveyzeh, lipid profile, liver enzymes, metabolic syndrome, Persian cohort, T2DM

## Abstract

**Introduction:**

Type 2 diabetes mellitus (T2DM) is among the world's top 10 leading causes of death. Additionally, prediabetes is a major risk factor for diabetes. Identifying diabetes co‐occurring disorders can aid in reducing adverse effects and facilitating early detection. In this study, we evaluated dyslipidaemia, metabolic syndrome (MetS), and liver enzyme levels in pre‐diabetic and T2DM patients in the Persian cohort compared to a control group.

**Materials and Methods:**

In this cross‐sectional study, 2259 pre‐diabetes, 1664 T2DM and 5840 controls (35–70 years) who were selected from the Hoveyzeh cohort centre were examined. Body mass index, blood pressure, fasting blood glucose (FBG), total cholesterol (TC), high‐density lipoprotein cholesterol (HDL‐C), triglyceride (TG) and liver enzymes: γ‐glutamyltransferase (GGT), alanine aminotransferase (ALT) and aspartate aminotransferase (AST) were determined using the standard protocols. MetS subjects were also identified based on the National Cholesterol Education Program guidelines.

**Results:**

Prediabetes and T2MD were closely correlated with the lipid profile, MetS, and liver enzymes (ALT, GGT, ALT/AST). MetS increases the risk of T2DM by 12.45 [95% CI: 10.88–14.24] fold, while an increase in ALT/AST ratio increases the risk of T2DM by 3.68 [95% CI: 3.159–4.154] fold. ROC curve analysis also revealed the diagnostic roles of GGT, ALT, AST and the ALT/AST ratio among pre‐diabetics, diabetics and the control group. The GGT level corresponds to the highest AUCs (0.685) with the highest sensitivity (70.25%).

**Conclusions:**

Our results indicated a significant increase in liver enzymes, lipid profile and MetS status in both pre‐diabetic and T2MD subjects, with the differences being more pronounced in diabetic individuals. Consequently, on the one hand, these variables may be considered predictive risk factors for diabetes, and on the other hand, they may be used as diagnostic factors. In order to confirm the clinical applications of these variables, additional research is required.

## Graphical abstract

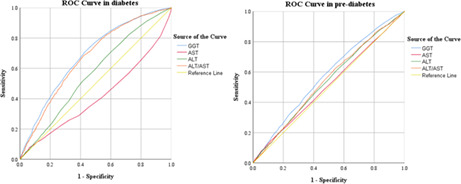

## 1 INTRODUCTION

Diabetes mellitus, as a metabolic disorder,^1^ is one of the most prevalent global public health issues^2^ and contributes to a rise in morbidity and mortality.^3^ According to estimates from the International Diabetes Federation (IDF), 1 in 11 individuals between the ages of 20 and 79 had type 2 diabetes mellitus (T2DM) in 2015,^4^ which could reach 629 million by 2045.^2^ Diabetes is hyperglycaemia resulting from insulin deficiency, insulin resistance or both.^1^, ^3^

Prediabetes is a major diabetes risk factor.^2^ It is a hyperglycaemic condition marked by impaired fasting glucose (IFG), impaired glucose tolerance (IGT) or glycated haemoglobin (A1C) of 6.0%–6.4%, or a combination of these.^1^, ^2^

Both dyslipidaemia and hypertension are significant risk factors for T2DM. According to the American Diabetes Association, patients with T2DM who have dysregulated levels of lipids such as total cholesterol, triglycerides, very‐low‐density lipoprotein (VLDL), low‐density lipoprotein (LDL) and high‐density lipoprotein (HDL) are diagnosed with diabetic dyslipidaemia. Alternatively, lipid markers may be a useful predictor of risk in diabetic patients.^5^

In addition, prediabetes and T2DM are common metabolic syndrome (MetS) manifestations.^1^ Some studies indicate that individuals with metabolic syndrome are four times more likely to develop T2DM.^6^ MetS are characterized by hypertriglyceridemia, low HDL cholesterol, abdominal obesity or a high BMI ratio, glucose intolerance or insulin resistance, hypertension and microalbuminuria.^7^

Insulin resistance syndrome may result in hepatic dysfunction, resulting in T2DM.^6^ Therefore, patients with advanced liver disease have a higher incidence of diabetes than the general population.^8^ Conversely, releasing free fatty acids (FFAs) due to T2DM decreases hepatic mitochondrial function. In turn, this causes further triglyceride storage in the hepatocyte and, ultimately, liver damage.^8^

Serum levels of liver enzymes, such as alanine aminotransferase (ALT), aspartate aminotransferase (AST), and to a lesser extent γ‐glutamyltransferase (GGT), are frequently used as indicators of liver damage.^9^ In the past decade, several studies have linked serum concentrations of these enzymes to multiple metabolic syndrome symptoms, including hepatic insulin resistance, T2DM and dyslipidaemia.^9^, ^10^, ^11^

Since then, little research has been conducted on the relationship between dyslipidaemia, metabolic syndrome and liver enzyme levels in pre‐diabetic and T2DM patients. In order to determine the relationship between these risk factors and the development of prediabetes and diabetes in the adult population of Hoveyzeh cohort centre, this study was conducted on three groups: healthy, pre‐diabetic and T2DM.

## 2 MATERIALS AND METHODS

We conducted a cross‐sectional study in men and women aged 35–70 who underwent a comprehensive health screening exam at the Hoveyzeh cohort centre for Prospective Epidemiological Research Studies in Iran (PERSIAN), a region in Iran's Southwest Khuzestan province, between 1 May 2016 and 31 August 2018. Therefore, 10,009 people were recruited in Hoveyzeh cohort centre. Patients with any of the following conditions at baseline were excluded from the study: a history of cancer, renal failure, known liver disease, ALT>3 times normal, alcohol consumption, recent (1 year) MI, acute coronary syndrome, stroke and weight loss of more than 5 kg in a month as well as microvascular complications. Finally, 9763 of 10,009 cases had the criteria for this study. All participants then completed questionnaires, including demographic information, cigarette smoking, opium use, consumable drugs, disease history and physical activity.

First, blood samples for analysis were obtained from the antecubital vein of patients and subjects who had fasted for 10 to 12 h. In the central laboratory of the Hoveyzeh cohort centre, all biochemical parameters were measured using standardized protocols on automated equipment.

Fasting serum glucose was assayed using the hexokinase/glucose‐6‐phosphate dehydrogenase method. Diabetes was defined as FBS levels ≥126 mg/dl or receiving anti‐diabetic drugs or self‐reported diagnosis of diabetes.

Standard enzymatic colorimetric techniques were used to measure serum total cholesterol (TC), triacylglycerol (TG) and high‐density lipoprotein cholesterol (HDL‐C) levels. The level of low‐density lipoprotein cholesterol (LDL‐C) was determined using the Friedewald et al. formula (LDL‐C = TC ‐ HDL ‐ VLDL cholesterol).^9^

The levels of AST, ALT and GGT were determined using the International Federation of Clinical Chemistry's method. All these analyses were done using commercial kits (Pars Azmon Inc.).

MetS is defined by three or more of the following National Cholesterol Education Program criteria: high TG (≥150 mg/dl); low HDL‐C (≤40 mg/dl) for men and <50 for women; high fasting blood sugar (≥100 mg/dl) or known type 2 diabetes; hypertension (at least 135/85 mmHg or receiving antihypertensive medication); and a waist circumference greater than 102 cm for men and 88 for women.^6^, ^12^, ^13^

### 2.1 Statistical analysis

The statistical analyses were conducted using SPSS (v. 15.0). For quantitative variables, data were presented as mean ± standard deviation; for qualitative variables, data are expressed as frequency (number (%)), The normality of data was determined using the Kolmogorov–Smirnov test, and the chi‐square test was used to determine the association between qualitative variables.

Differences between the two groups were calculated by Mann–Whitney tests for skewed data. In addition, the Kruskal–Wallis test was used to compare variables in three groups.

Moreover, logistic regression analysis was employed to calculate studied risk factors for prediabetes and diabetes vs. control group. Then, multivariable model was performed for adjusting of age, gender and BMI. Receiver operating characteristic (ROC) curve analysis was used to determine the prognostic relationship of liver enzymes and lipid profile in prediabetes and diabetes. All *p*‐values were two‐tailed, and *p* < .05 were considered statistically significant.

## 3 RESULTS

### 3.1 Characteristics of the study participants according to FBS tertiles

The final database contained 9763 subjects (3809 males and 5954 females); subjects were divided into three groups based on FBS levels. Table 1 illustrates the characteristics of three distinct groups. T2DM prevalence was 17.0% (18.1% in males and 16.4% in females), prediabetes prevalence was 23.1% (21.0% in males and 24.5% in females), and control prevalence was 59.8% (60.9% in males, 59.1% in females). Participants with prediabetes and T2DM were older and had a higher BMI, waist circumference, diastolic blood pressure (DBP) and systolic blood pressure (SBP) than control subjects.

**TABLE 1:** Anthropometrics and biochemical characteristics of the study participants according to the tertiles of FBS

Variables	Fasting glucose (mg/dl)			
	FBS ≤100 5840 (59.82%)	FBS: 100–125 2259 (23.14%)	FBS≥126 1664 (17.04%)	*p*‐Value
Anthropometrics				
Gender				
Male	2321 (60.9%) ^A^	799 (21.0%) ^B^	689 (18.1%) ^C^	<.0001**
Female	3519 (59.1%) ^A^	1460 (24.5%) ^B^	975 (16.4%) ^C^	
Age (year)	47.03 ± 8.79 ^A^	50.53 ± 9.27 ^B^	52.81 ± 8.89 ^C^	<.0001*
Waist Circumference (cm)	97.62 ± 11.83 ^A^	102.45 ± 12.09 ^B^	103.15 ± 11.46 ^B^	<.0001*
BMI (kg/m^2^)	28.14 ± 5.15 ^A^	30.03 ± 5.52 ^B^	29.63 ± 5.23 ^C^	<.0001*
Diastolic blood pressure (mmHg)	70.32 ± 10.97 ^A^	72.60 ± 11.38 ^B^	73.16 ± 11.51 ^B^	<.0001*
Systolic blood pressure (mmHg)	110.46 ± 16.95^A^	115.49 ± 18.90 ^B^	118.27 ± 20.19 ^C^	<.0001*
Metabolic syndrome				
No	4409 (75.5%) ^A^	630 (27.9%) ^B^	330 (19.8%) ^C^	<.0001**
Yes	1413 (24.5%) ^A^	1629 (72.1) ^B^	1334 (80.2%) ^C^	
Biochemicals				
FBS (mg/dl)	88.97 ± 6.54 ^A^	108.25 ± 6.96 ^B^	201.48 ± 67.98 ^C^	<.0001*
LDL (mg/dl)	105.62 ± 31.17^A^	109.51 ± 33.83^A^	106.86 ± 37.33^B^	<.0001*
TG (mg/dl)	147.6 ± 84.2 ^A^	170.06 ± 107.3 ^B^	202.02 ± 135.4 ^C^	<.0001*
Total Cholesterol (mg/dl)	185.76 ± 37.1 ^A^	193.97 ± 40.8 ^B^	196.34 ± 47.9 ^B^	<.0001*
HDL (mg/dl)	50.68 ± 12.24 ^A^	50.34 ± 11.75 ^A^	49.21 ± 11.61 ^B^	<.0001*
Hepatic enzymes				
AST (units/L)	18.06 ± 7.62 ^A^	19.30 ± 9.19 ^A^	17.36 ± 9.05 ^B^	<.0001*
ALT (units/L)	20.52 ± 13.72 ^A^	22.03 ± 14.88 ^B^	22.25 ± 13.35 ^C^	<.0001*
GGT (units/L)	24.14 ± 16.61 ^A^	27.05 ± 17.68 ^B^	34.73 ± 34.01 ^C^	<.0001*
ALT/AST	1.06 ± 0.384 ^A^	1.10 ± 0.380 ^B^	1.28 ± 0.514 ^C^	<.0001*

*Note*: The total 9763 subjects were divided into tertiles according to FBS distribution and the significance of any differences in means or proportions were tested with analysis of Kruskal–Wallis* and chi‐squared** tests, respectively. The results are presented as the means ± SD. Similar letters (A, B and C) indicate that there is no significant difference between the mean in the groups (*p*‐value > .05). Different letters (A, B and C) indicate that there is a significant difference between the mean in the groups (*p*‐value < .05).

Abbreviations: ALT, Alanine aminotransferase; AST, Aspartate aminotransferase; BMI, Body mass index; FBS, Fasting blood sugar; GGT, Gamma‐glutamyltransferase; HDL, High density lipoprotein; LDL, Low density lipoprotein.

In prediabetes and T2DM, biochemical variables, including TG, were significantly higher than in the control group. Compared to the control group, prediabetes and diabetes had significantly higher mean total cholesterol levels, whereas there was no significant difference between prediabetes and diabetes. In addition, the mean LDL in diabetes and normal groups was significantly higher than in the prediabetes group, but there was no significant difference between diabetes and normal groups. In contrast, the HDL level was significantly lower in T2DM compared to prediabetes and the control group, whereas there was no significant difference between prediabetes and the control group.

Those who developed prediabetes and T2DM had significantly higher levels of hepatic enzymes, including GTT and ALT, compared to the control group. In contrast, the mean AST was significantly lower in T2DM than in prediabetes and the control group, and there was no significant difference between prediabetes and the control group (Table 1).

### 3.2 ROC curve analysis

**FIGURE 1:**
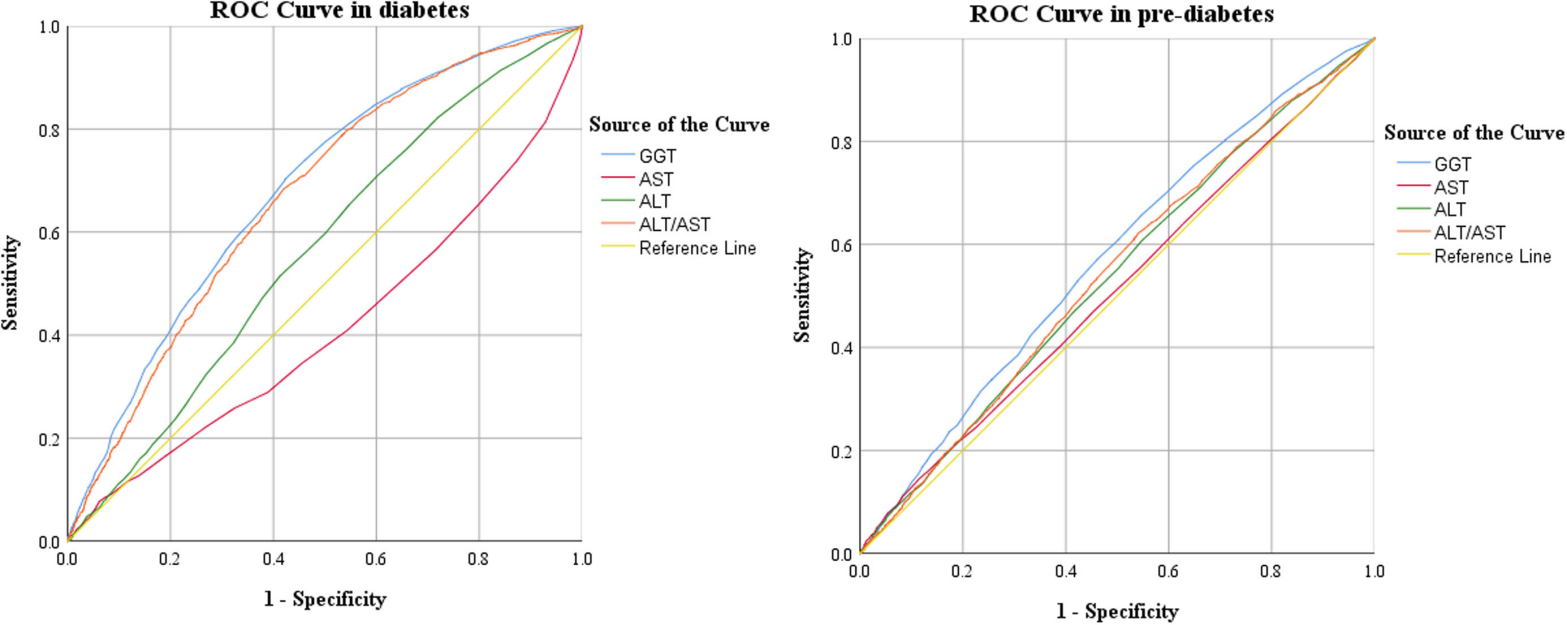
Receiver operating characteristic curve analysis of GGT, ALT, AST and ALT/AST ratio in diabetes and pre‐diabetes (data from Table 2).

Receiver operating characteristic curve analysis revealed the significance of GGT, ALT, AST and the ALT/AST ratio in identifying prediabetes or diabetes (Table 2, Figure 1). The ROC curve analysis is presented in Table 2.

**TABLE 2:** Receiver operating characteristic curve analysis of GGT, ALT, AST and ALT/AST ratio in diabetes and pre‐diabetes, respectively

Variable	AUC	95% CI	*p*‐Value	Cut‐off value	Sensitivity(%)	Specificity(%)
Diabetes						
GGT	0.685	0.673–0.694	<0.0001	21.36	70.25%	57.76%
ALT	0.564	0.553–0.575	<0.0001	14	70.85%	39.88%
AST	0.412	0.395–0.428	<0.0001	14	43.33%	71.64%
ALT/AST	0.669	0.658–0.679	<0.0001	1.06	68.87%	57.29%
Pre‐diabetes						
GGT	0.573	0.562–0.583	<0.0001	20.33	57.10%	53.89%
ALT	0.537	0.526–0.548	<0.0001	15	60.69%	45.26%
AST	0.513	0.502–0.523	0.082	25	14.52%	88.48%
ALT/AST	0.542	0.531–0.553	<0.0001	0.95	61.66%	46.25%

*Note*: Data were reported as area under curve (AUC) (95% confidence interval).

Roc curve analysis of GTT, ALT, AST and ALT/AST in diabetes vs. control group was as follows: AUC = 0.685; (95% CI: 0.673–0.694; *p* < .0001; Cut‐off value: >21.36; Sensitivity: 70.25%; Specificity: 57.76%), AUC = 0.564; (95% CI: 0.553–0.575; *p* < .0001; Cut‐off value: >14; Sensitivity: 70.85%; Specificity: 39.88%), AUC = 0.588; (95% CI: 0.577–0.600; *p* < .0001; Cut‐off value: <14; Sensitivity: 43.33%; Specificity: 71.64%), AUC = 0.669; (95% CI: 0.658–0.679; *p* < .0001; Cut‐off value: >1.06; Sensitivity: 68.87%; Specificity: 57.29%), respectively. Similar results were also observed in the case of prediabetes vs. control group, including: AUC = 0.573; (95% CI: 0.562–0.583; *p* < .0001; Cut‐off value: >20.33; Sensitivity: 57.10%; Specificity: 53.89%), AUC = 0.537; (95% CI: 0.526–0.548; *p* < .0001; Cut‐off value:>15; Sensitivity: 60.69%; Specificity: 45.26%), AUC = 0.513; (95% CI: 0.526–0.548; *p* = .082; Cut‐off value: >25; Sensitivity: 14.52%; Specificity: 88.48%), AUC = 0.542; (95% CI: 0.531–0.553; *p* < .0001; Cut‐off value: >0.95; Sensitivity: 61.66%; Specificity: 46.25%).

### 3.3 Logistic regression analysis

According to logistic regression analysis, some liver enzymes, lipid profiles and metabolic syndrome were associated with an increased odds of developing prediabetes or diabetes (Table 3). The estimated ORs for metabolic syndrome in the prediabetes and diabetes groups were 7.966 (95% CI: 7.139–8.889; *p* < .0001) and 12.45 (95% CI: 10.88–14.24; *p* < .0001), respectively. In the case of AST, however, the odds ratio (0.976) indicated a reduction in diabetes odds (95% CI: 0.968–0.984; *p* < .0001). On the contrary, the ALT/AST ratio increases the odds of prediabetes and diabetes development by 1.347 (95% CI: 0.968–0.984; *p* < .0001) and 3.623 (95% CI: 3.159–4.154; *p* < .0001, respectively). After the adjustment for age, sex and BMI, there was almost no difference with the results obtained from univariate analysis. However, both analyses display significantly positive relationships between ALT/AST ratio and metabolic syndrome with prediabetes and diabetes.

**TABLE 3:** Logistic regression analysis of liver enzymes, lipid profile, and MetS in prediabetes and diabetes vs. control group

Prediabetes				Diabetes		
Variable	Odds Ratios	95% CI	*p*‐Value	Odds Ratios	95% CI	*p*‐Value
model 1^a^						
GGT	1.009	1.006–1.012	<.0001	1.024	1.021–1.027	<.0001
AST	1.010	1.004–1.016	<.0001	0.976	0.968–0.984	<.0001
ALT	1.007	1.004–1.010	<.0001	1.008	1.004–1.012	<.0001
ALT/AST	1.347	1.190–1.525	<.0001	3.623	3.159–4.154	<.0001
LDL	1.003	1.002–1.005	<.0001	1.001	0.999–1.002	<.0001
HDL	0.997	0.993–1.001	<.0001	0.989	0.984–0.993	<.0001
TG	1.002	1.001–1.002	<.0001	1.005	1.004–1.005	<.0001
TC	1.005	1.004–1.006	<.0001	1.006	1.005–1.007	<.0001
MetS	7.966	7.139–8.889	<.0001	12.45	10.88–14.24	<.0001
model 2^b^						
GGT	1.010	1.007–1.013	<.0001	1.024	1.021–1.027	<.0001
AST	1.015	1.009–1.021	<.0001	0.979	0.970–0.988	<.0001
ALT	1.012	1.009–1.016	<.0001	1.014	1.010–1.018	<.0001
ALT/AST	1.595	1.382–1.842	<.0001	5.632	4.776–6.640	<.0001
LDL	1.002	1.000–1.003	.040	0.999	0.997–1.000	.151
HDL	0.996	0.992–1.001	.087	0.988	0.983–0.993	<.0001
TG	1.002	1.002–1.003	<.0001	1.005	1.004–1.006	<.0001
TC	1.004	1.002–1.005	<.0001	1.005	1.003–1.006	<.0001
MetS	6.833	6.100–7.654	<.0001	10.67	9.268–12.28	<.0001

^a^ Model 1: Crude model.

^b^ Model 2: Adjusted for age, gender and body mass index (BMI).

Abbreviation: CI, confidence interval.

## 4 DISCUSSION

In the current study, we observed a significant increase in all metabolic risk factors and liver enzymes, except for HDL‐C and AST, in both prediabetic and T2MD subjects, with the differences being more pronounced in diabetic individuals.

In subjects with prediabetes and T2DM, the mean LDL, TG and TC levels were higher. Consistent with these findings, Dhoj et al.^14^ demonstrated that diabetes is associated with a high prevalence of dyslipidaemia characterized by elevated levels of cholesterol, TG and LDL. Additionally, Jasim et al.^5^ identified TG as one of the promising biomarkers for predicting prediabetes and T2DM. These findings support that diabetes patients are more susceptible to co‐occurring diseases such as hyperglycaemia, chronic renal failure, hypothyroidism and polypharmacy, with drugs known to have adverse effects on lipid profiles. Patients with diabetes must therefore be treated to prevent coronary artery disease.^15^

Individual metabolic syndrome characteristics (such as higher BMI, waist circumference, DBP and SBP levels, among others) were associated with the prevalence of prediabetes and T2DM, according to the findings of this study. Thus, 80% of subjects with T2DM and 72% in the prediabetes group had MetS, whereas only 24% of the control group exhibited metabolic syndrome symptoms. In addition, Ogedengbe et al.^16^ found that the prevalence of MetS among T2DM patients is extremely high.

This study revealed that liver enzymes, including ALT and GGT but not AST, and the ALT/AST ratio were significantly elevated in prediabetes and T2MD cases. However, some studies have found no correlation between elevated ALT and diabetes, possibly due to the ethnic diversity of the study populations.^6^ Forlani et al.^17^ reported a high prevalence of elevated ALT, AST and GGT levels in T2DM, which is consistent with our findings. Although there are no clear biological explanations for the relationships between liver indicators and glucose metabolism, one possible mechanism is that MetS and T2DM increase the risk of liver damage, increasing liver enzyme levels.^9^ To reduce the risk of liver damage, prediabetics and diabetic patients may require a comprehensive clinical, laboratory and histological examination.

In addition, GGT, ALT and the ALT/AST ratio, but not AST, can be used to identify prediabetes and diabetes based on ROC results. Among prediabetic and diabetic subjects, the GGT level has the highest areas under the curve (AUC) and the highest sensitivity compared to the control group. In contrast, logistic regression analysis revealed that higher levels of ALT, GGT and ALT/AST were independent risk factors for prediabetics and diabetics and that an increase in the ALT/AST ratio increased the risk of T2MD by 3.68‐fold, whereas lower AST levels were associated with the risk of diabetes. Sun‐Hye et al.^18^ observed that higher levels of GGT and ALT and a lower AST/ALT ratio were independent risk factors for diabetes and impaired fasting glucose (IFG). Additionally, Zhao et al.^19^ evidenced that the ALT/AST ratio may be a useful indicator of insulin resistance (IR) in the Chinese population.

According to several studies, elevated GGT and ALT levels are also beneficial for identifying early markers of dysregulated glucose metabolism, which strongly correlate with prediabetes and diabetes.^20^ A second proposed mechanism for the relationship between hepatic indices and glucose metabolism is that elevated serum ALT and GGT levels indicate hepatic steatosis, resulting in hepatic insulin resistance (IR).^18^ IR is a risk factor for T2DM.^19^ Therefore, it is unknown whether T2DM increases liver enzyme levels or whether elevated liver enzyme levels increase the risk of developing T2DM. Therefore, additional research is required to clarify these theories.

In contrast to our findings, some studies have found that elevated GGT levels, but not ALT or AST, can be used to predict the onset of T2DM.^9^ Sattar et al.^21^ also demonstrated that elevated ALT levels within the ‘normal’ range predict diabetes independently of elevated AST levels. Although we did not examine the role of gender in transaminase levels in this study, a possible explanation for these contradictory findings may be that transaminase levels are gender‐specific, according to the findings of some studies.^22^ Consequently, it appears that using the ratio of variables, such as ALT/AST, rather than each variable individually may be more effective in evaluating diabetes patients.

## 5 CONCLUSION

Our results indicated a significant increase in liver enzymes except AST, lipid profile except HDL‐C, and MetS status in both prediabetic and T2MD subjects, with the differences being more pronounced in diabetic individuals. On the one hand, these variables or their ratio may be considered predictive risk factors for diabetes, and on the other hand, they may be utilized as diagnostic factors. However, it is unknown whether T2DM increases liver enzyme levels or whether elevated liver enzyme levels increase the incidence of T2DM, and the pathophysiologic pathways underlying this association are unclear. Therefore, additional research is required to clarify these theories and validate their clinical applications.

## AUTHOR CONTRIBUTIONS

N. M. designed and supervised the study. N. D. wrote the paper. S. SP., Z. R. and B. C. analysed data. All authors read and approved the final manuscript.

## CONFLICT OF INTEREST

The authors declare no conflict of interest.

## ETHICAL APPROVAL

This study was approved by the Ethics Committee of Ahvaz Jundishapur University of Medical Sciences (Ethical code: IR. AJUMS. REC.1398.455), and the informed consent was taken from all patients who participated in Hoveyzeh Cohort.

## Data Availability

Data will be made available on request.

## References

[1] Punthakee Z , Goldenberg R , Katz P . Definition, classification and diagnosis of diabetes, prediabetes and metabolic syndrome. Can J Diabetes. 2018;42:S10‐S15. 29650080 10.1016/j.jcjd.2017.10.003

[2] Zhao K , Yang S‐S , Wang H‐B , Chen K , Lu Z‐H , Mu Y‐M . Association between the hypertriglyceridemic waist phenotype and prediabetes in Chinese adults aged 40 years and older. J Diabetes Res. 2018;2018:1‐9. 10.1155/2018/1031939 PMC6036789 30046615

[3] Takahashi M , Okimura Y , Iguchi G , et al. Chemerin regulates β‐cell function in mice. Sci Rep. 2011;1(1):1‐10. 22355640 10.1038/srep00123 PMC3216604

[4] Zheng Y , Ley SH , Hu FB . Global aetiology and epidemiology of type 2 diabetes mellitus and its complications. Nat Rev Endocrinol. 2018;14(2):88‐98. 29219149 10.1038/nrendo.2017.151

[5] Jasim OH , Mahmood MM , Ad'hiah AH . Significance of lipid profile parameters in predicting pre‐diabetes. Arch Razi Inst. 2022;77(1):267‐274. 10.22092/ARI.2021.356465.1846 PMC9288615 35891716

[6] Wannamethee SG , Shaper AG , Lennon L , Whincup PH . Hepatic enzymes, the metabolic syndrome, and the risk of type 2 diabetes in older men. Diabetes Care. 2005;28(12):2913‐2918. 16306554 10.2337/diacare.28.12.2913

[7] Qian Y , Lin Y , Zhang T , et al. The characteristics of impaired fasting glucose associated with obesity and dyslipidaemia in a Chinese population. BMC Public Health. 2010;10(1):1‐8. 20233452 10.1186/1471-2458-10-139 PMC2851684

[8] Bell DS , Allbright E . The multifaceted associations of hepatobiliary disease and diabetes. Endocr Pract. 2007;13(3):300‐312. 17599864 10.4158/EP.13.3.300

[9] Music M , Dervisevic A , Pepic E , et al. Metabolic syndrome and serum liver enzymes level at patients with type 2 diabetes mellitus. Med Arch. 2015;69(4):251‐255. 26543313 10.5455/medarh.2015.69.251-255 PMC4610622

[10] Kramer CK , Retnakaran R . Liver enzymes and type 2 diabetes: a complex two‐way relationship. J Diabetes Complications. 2013;27(4):301‐302. 23684419 10.1016/j.jdiacomp.2013.04.009

[11] Rector RS , Thyfault JP , Wei Y , Ibdah JA . Non‐alcoholic fatty liver disease and the metabolic syndrome: an update. World J Gastroenterol: WJG. 2008;14(2):185‐192. 18186553 10.3748/wjg.14.185 PMC2675112

[12] Saki N , Karandish M , Cheraghian B , Heybar H , Hashemi SJ , Azhdari M . Prevalence of cardiovascular diseases and associated factors among adults from Southwest Iran: baseline data from Hoveyzeh cohort study. BMC Cardiovasc Disord. 2022;22(1):1‐10. 35804295 10.1186/s12872-022-02746-y PMC9270737

[13] Ghaedrahmat Z , Cheraghian B , Jaafarzadeh N , Takdastan A , Shahbazian HB , Ahmadi M . Relationship between urinary heavy metals with metabolic syndrome and its components in population from Hoveyzeh cohort study: A case‐control study in Iran. J Trace Elem Med Biol. 2021;66:126757. 33839459 10.1016/j.jtemb.2021.126757

[14] Thapa SD , SR KC , Gautam S , Gyawali D . Dyslipidemia in type 2 diabetes mellitus. J Pathol Nepal. 2017;7(2):1149‐1154.

[15] Chehade JM , Gladysz M , Mooradian AD . Dyslipidemia in type 2 diabetes: prevalence, pathophysiology, and management. Drugs. 2013;73(4):327‐339. 23479408 10.1007/s40265-013-0023-5

[16] Ogedengbe S , Ezeani I , Aihanuwa E . Comparison of clinical and biochemical variables in type 2 diabetes mellitus patients and their first‐degree relatives with metabolic syndrome in Benin City, Nigeria: a cross sectional case controlled study. Endocr Regul. 2016;50(1):32‐40. 27560635 10.1515/enr-2016-0007

[17] Forlani G , Di Bonito P , Mannucci E , et al. Prevalence of elevated liver enzymes in type 2 diabetes mellitus and its association with the metabolic syndrome. J Endocrinol Invest. 2008;31(2):146‐152. 18362506 10.1007/BF03345581

[18] Ko S‐H , Baeg MK , Han K‐D , Ko S‐H , Ahn Y‐B . Increased liver markers are associated with higher risk of type 2 diabetes. World J Gastroenterol: WJG. 2015;21(24):7478‐7487. 26139993 10.3748/wjg.v21.i24.7478 PMC4481442

[19] Zhao L , Cheng J , Chen Y , et al. Serum alanine aminotransferase/aspartate aminotransferase ratio is one of the best markers of insulin resistance in the Chinese population. Nutr Metab (Lond). 2017;14(1):1‐9. 29051770 10.1186/s12986-017-0219-x PMC5633891

[20] Rückert I‐M , Heier M , Rathmann W , Baumeister SE , Döring A , Meisinger C . Association between markers of fatty liver disease and impaired glucose regulation in men and women from the general population: the KORA‐F4‐study. PLoS One. 2011;6(8):e22932. 21850244 10.1371/journal.pone.0022932 PMC3151286

[21] Sattar N , Scherbakova O , Ford I , et al. Elevated alanine aminotransferase predicts new‐onset type 2 diabetes independently of classical risk factors, metabolic syndrome, and C‐reactive protein in the west of Scotland coronary prevention study. Diabetes. 2004;53(11):2855‐2860. 15504965 10.2337/diabetes.53.11.2855

[22] Buday B , Pach PF , Literati‐Nagy B , et al. Sex influenced association of directly measured insulin sensitivity and serum transaminase levels: why alanine aminotransferase only predicts cardiovascular risk in men? Cardiovasc Diabetol. 2015;14(1):1‐13. 25986611 10.1186/s12933-015-0222-3 PMC4492083

